# Supplementing Ca salts of soybean oil to late-gestating beef cows: impacts on performance and physiological responses of the offspring

**DOI:** 10.1093/tas/txaa090

**Published:** 2020-12-22

**Authors:** Alice Poggi Brandão, Reinaldo F Cooke, Kelsey M Schubach, Bruna Rett, Osvaldo A Souza, Ky G Pohler, David W Bohnert, Rodrigo S Marques

**Affiliations:** 1 Texas A&M University, Department of Animal Science, College Station, TX; 2 Universidade Estadual Paulista—FMVZ, Botucatu, Brazil; 3 Oregon State University—EOARC, Burns, OR; 4 Montana State University, Department of Animal and Range Sciences, Bozeman, MT

## INTRODUCTION

Nutritional management of beef cows during late gestation has direct implications on postnatal performance of the in utero offspring (Cooke, 2019). The majority of the research conducted within this subject focused on energy and protein nutrition, and limited information exists about essential fatty acids (FA). Our group recently reported that supplementing Ca salts of ɷ-3 and ɷ-6 FA to beef cows during late gestation improved offspring performance (Marques et al., 2017). More specifically, feedlot growth rate and carcass marbling were greater in calves born from cows supplemented with ɷ-3 + ɷ-6 FA. These results were indicative of programming effects from essential FA supplementation, by improving hyperplastic muscle development and intramuscular adipose cells of calves during gestation.

The mechanisms underlying the outcomes reported by Marques et al. (2017) still warrant investigation, including the individual role of ɷ-3 and ɷ-6 FA. In recent studies with beef cattle, linoleic acid and its ɷ-6 derivatives were the specific FA linked with fetal cell differentiation and development (Schubach et al., 2019). Hence, supplementing a FA source based on ɷ-6 such as Ca salts of soybean oil (CSSO), may be more advantageous to gestational programming compared with the ɷ-3 + ɷ-6 FA mix used by Marques et al. (2017), and also elucidate the specific programming roles of ɷ-6 FA. Based on this rationale, we hypothesized that CSSO supplementation to late-gestating beef cows will improve postnatal offspring productivity via programming effects. This experiment compared growth, physiological parameters, and carcass characteristics of cattle born from cows supplemented or not with CSSO during the last trimester of gestation.

## MATERIALS AND METHODS

This experiment was conducted at the Oregon State University—EOARC Burns, and approved by the Oregon State University—Animal Care and Use Committee (#4974).

### Cow–Calf Management and Dietary Treatments

Nonlactating, pregnant, multiparous Angus × Hereford cows (*n* = 104) were assigned to this experiment at the end of their 2nd trimester of gestation. Cows conceived during the same fixed-time artificial insemination protocol (Marques et al., 2017) using semen from two Angus sires (d 195 of gestation on d 0).

Prior to the beginning of the experiment (d −15), cows were ranked by sire, body weight (BW), and body condition score (BCS), and randomly assigned to receive (dry matter basis) 415 g of soybean meal per cow daily in addition to 1) 195 g/cow daily of CSSO (Essentiom; Church and Dwight Co., Inc., Princeton, NJ; CSSO, *n* = 52) or 2) 170 g/cow daily of prilled saturated fat (EnergyBooster, Milk Specialties, Eden Prairie, MN) + 25 g/cow daily of limestone (CON, *n* = 52). Supplement treatments were iso-nitrogenous, iso-lipidic, and iso-caloric (Table 1), and provided from d 0 to calving. Cows were maintained in one of two meadow foxtail (*Alopecurus pratensis* L.) pastures (52 cows/pasture, 26 cows/treatment per pasture) from d −15 until calving. Grass-alfalfa hay was provided daily at 12.7 kg/cow (dry matter basis), and cows had ad libitum access to water and a commercial mineral + vitamin mix.

**Table 1.** Composition and nutritional profile of diets containing Ca salts of soybean oil (CSSO) or prilled saturated fat (CON)

Item	CON	CSSO
Ingredients, kg/d dry matter basis		
Grass-alfalfa hay	12.7	12.7
Soybean meal	0.415	0.415
Essentiom^*a*^	0	0.195
EnergyBooster^*b*^	0.170	0
Limestone	0.025	0
Nutrient profile,^*c*^ dry matter basis		
Dry matter, %	91.9	92.1
Net energy for maintenance,^*c*^ Mcal/kg	1.28	1.28
Crude protein, %	8.3	8.3
Fatty acids, %	2.46	2.45
Palmitic (16:0), %	0.64	0.63
Stearic (18:0), %	0.61	0.08
Oleic (18:1), %	0.16	0.41
Linoleic (18:2), %	0.29	0.65
Linolenic (18:3), %	0.31	0.35

^*a*^Essentiom (Church and Dwight Co., Inc., Princeton, NJ).

^*b*^Energy Booster 100 (Milk Specialties, Eden Prairie, MN).

^*c*^Values obtained via wet chemistry analysis (Dairy One Forage Laboratory, Ithaca, NY).

From d 0 until calving, cows were gathered three times weekly (Mondays, Wednesdays, and Fridays) and sorted into 1 of 24 individual feeding pens. Cows individually received treatments (1.42 kg of supplement treatment/feeding, dry matter basis). Immediately after calving, cow–calf pairs were removed from their pasture and assigned to the general management of the research herd until weaning (Marques et al., 2017), which did not include CSSO or CON supplementation.

Calves were weaned on d 290 of the experiment, and transferred to a 6-ha meadow foxtail pasture for a 35-d preconditioning period as a single group. On d 325, all calves were loaded into a commercial livestock trailer and transported for 215 km to commercial feedlot (Cannon Hill Feeders LLC., Nyssa, OR), where they remained as a single group until slaughter (Agri Beef Co., Toppenish, WA). Calves did not receive supplemental CSSO or CON during preconditioning or in the feedlot.

### Sampling

Samples of all ingredients fed to late-gestating cows were collected before the beginning of the experiment and analyzed for nutrient and FA content (Dairy One Forage Laboratory, Ithaca, NY). Cow BW and BCS were recorded and a blood sample was collected prior to the beginning of the experiment (d −15). Within 12 h after calving, cow BW, cow BCS, calf birth BW, and calf gender were recorded, and blood was collected from cows and calves. Blood samples were collected, processed, and analyzed for plasma FA concentration as in Schubach et al. (2019).

At weaning (d 290), cow BW and BCS were recorded. Calf BW was recorded over 2 consecutive days upon weaning (d 290 and 291) and prior to shipping (d 324 and 325), which were averaged to calculate preconditioning BW gain. From weaning until slaughter, calves were observed daily for bovine respiratory disease (BRD) signs and treated when signs were observed (Marques et al., 2017). Carcass traits were collected upon slaughter at the commercial packing plant, including hot carcass weight (HCW) that was adjusted to a 63% dressing percentage to estimate final BW and feedlot BW gain.

### Statistical Analysis

All variables were analyzed with cow as the experimental unit, and cow(treatment × pasture), pasture, and sire as random variables using SAS (SAS Inst. Inc., Cary, NC). Quantitative data were analyzed using the MIXED procedure, and binary data were analyzed using the GLIMMIX procedure. Model statements for cow-related responses included the effects of treatment. Model statements for calf-related responses analysis included the effects of treatment, calf sex, and the treatment × calf sex interaction. Results are reported as least square means. Significance was set at *P* ≤ 0.05.

## RESULTS AND DISCUSSION

### Cow Parameters

Composition and nutritional profile of diets (hay + treatment) offered to CSSO- and CON-supplemented cows are described in Table 1. No treatment effects were detected (*P* ≥ 0.20) for cow BW and BCS (Table 2). Plasma FA profile did not differ (*P* ≥ 0.19) between CSSO and CON cows on d −15 (data not shown), indicating similar FA profile before treatment administration. At calving, CSSO cows had greater (*P* < 0.01) plasma concentrations of linoleic acid and total ω-6 FA compared with CON (Table 3). These results are in accordance with the FA content and intake of treatments, given that plasma profile reflects intake and intestinal FA flow (Hess et al., 2008).

**Table 2.** Performance of beef cows receiving diets containing Ca salts of soybean oil (CSSO; *n* = 52) or prilled saturated fat (CON; *n* = 52) during the last trimester of gestation^*a*,*b*^

Item	CON	CSSO	SEM	*P*
Days receiving treatments, d	85.5	85.1	0.6	0.60
Body weight, kg				
Initial	504	505	9	0.89
Calving	545	554	9	0.48
Weaning	568	564	9	0.78
Body condition score				
Initial	4.88	4.88	0.04	0.92
Calving	4.74	4.82	0.07	0.59
Weaning	5.19	5.09	0.08	0.40

^*a*^Cows received (dry matter basis) 190 g/cow daily of Ca salts of soybean oil (CSSO; Essentiom; Church and Dwight Co., Inc., Princeton, NJ) or 170 g/cow daily of prilled saturated fat (CON; Energy Booster 100; Milk Specialties, Eden Prairie, MN). Treatments were provided from d 0 (d 195 of gestation) until calving.

^*b*^Values recorded prior to the beginning of the experiment (initial; d −15), within 12 h of after calving, and at weaning (d 290).

**Table 3.** Plasma FA profile (µg/mL of plasma) of beef cows and their offspring upon calving^*a*,*b*^

Item	CON	CSSO	SEM	*P*-value
Linoleic (18:2 n-6)				
Calf	24.5	41.8	4.3	<0.01
Cow	145	341	11	<0.01
Linolenic (18:3 n-3)				
Calf	1.23	0.100	0.285	<0.01
Cow	60.5	34.2	1.5	<0.01
Total polyunsaturated fatty acid				
Calf	40.1	60.6	4.6	<0.01
Cow	270	450	15	<0.01
Total ω-3				
Calf	2.50	1.05	0.55	0.05
Cow	67.4	41.7	1.7	<0.01
Total ω-6				
Calf	37.6	59.5	4.4	<0.01
Cow	203	408	13	<0.01
Total identified FA				
Calf	311	319	15	0.73
Cow	575	752	25	<0.01

Cows received diets containing Ca salts of soybean oil (CSSO; *n* = 52) or prilled saturated fat (CON; *n* = 52) during the last trimester of gestation.

^*a*^Cows received (dry matter basis) 190 g/cow daily of Ca salts of soybean oil (CSSO; Essentiom; Church and Dwight Co., Inc., Princeton, NJ) or 170 g/cow daily of prilled saturated fat (CON; Energy Booster 100; Milk Specialties, Eden Prairie, MN). Treatments were provided from d 0 (d 195 of gestation) until calving.

^*b*^Blood samples were collected from cows and calves within 12 h of after calving.

### Offspring Responses

No treatment differences were detected (*P* ≥ 0.36) for calf birth BW and proportion of male calves born (Table 4). Calves from CSSO cows also had greater (*P* < 0.01) plasma concentrations of linoleic acid and total ω-6 FA compared with CON cohorts, corroborating differences noted between CSSO and CON cows given that maternal circulating FA are transferred to the fetus via placenta (Marques et al., 2017). No treatment differences in calf responses were noted (*P* ≥ 0.17), not BRD signs were observed until the end of preconditioning (Table 4). Others have also reported similar birth and weaning BW, and well as preconditioning performance in calves from cows supplemented or not with ω-6 FA during gestation (Hess et al., 2008; Marques et al., 2017).

**Table 4.** Calving, weaning, and preconditioning responses from offspring of beef cows receiving diets containing Ca salts of soybean oil (CSSO; *n* = 52) or prilled saturated fat (CON; *n* = 52) during the last trimester of gestation^*a*^

Item	CON	CSSO	SEM	*P*-value
Calving results				
% of male calves born	56.6	51.0	7.0	0.57
Calf birth weight, kg	37.0	37.7	0.6	0.42
Adjusted calf birth weight,^*b*^ kg	38.3	39.1	0.6	0.36
Weaning results				
Weaning rate, %	96.0	100	2.0	0.17
% of male calves weaned	56.9	51.0	7.0	0.56
Calf weaning age, d	209	209	0.1	0.91
Calf weaning weight, kg	262	264	4	0.72
Calf 205-d adjusted weaning weight,^*b*^ kg	267	270	4	0.57
Preconditioning results				
Average daily gain, kg/d	0.68	0.67	0.05	0.84
Final BW, kg	287	289	4	0.77

^*a*^Cows received (dry matter basis) 190 g/cow daily of Ca salts of soybean oil (CSSO; Essentiom; Church and Dwight Co., Inc., Princeton, NJ) or 170 g/cow daily of prilled saturated fat (CON; Energy Booster 100; Milk Specialties, Eden Prairie, MN). Treatments were provided from d 0 (d 195 of gestation) until calving.

^*b*^According to BIF (2010).

As designed, days in the feedyard were similar (*P* = 0.99) between calves from CSSO and CON cows (Table 5). Overall BRD incidence was similar (*P* = 0.16) between treatments (Table 5). Nonetheless, the incidence of calves diagnosed with BRD that required a second antimicrobial treatment was less (*P* = 0.03) in calves from CSSO cows, resulting in reduced (*P* = 0.05) need of treatments compared with CON (Table 5). Treatment × calf sex interactions were detected (*P* ≤ 0.03) for feedlot average daily gain, final BW, and HCW. These responses were greater (*P* ≤ 0.05) in steers from CSSO cows compared with CON (Table 5), and did not differ (*P* ≥ 0.59) between heifers (Table 5). A treatment effect was detected (*P* = 0.03) for carcass longissimus muscle (LM) area, which was greater in calves from CSSO cows compared with CON across sexes (Table 5). No other treatment effects were noted (*P* ≥ 0.43) for carcass traits (Table 5).

**Table 5.** Feedlot performance and carcass characteristics from offspring of beef cows receiving diets containing CSSO (*n* = 52) or prilled saturated fat (CON; *n* = 52) during the last trimester of gestation^*a*^

Item	CON	CSSO	SEM	*P*-value
Days on feed, d	188	188	0.2	0.99
Cattle treated for respiratory disease, %				
Once	40.5	28.4	6.7	0.16
Twice	19.2	5.64	4.79	0.03
Number of antimicrobial treatments required	1.49	1.18	0.10	0.05
Average daily gain, kg/d				
Steers	1.39	1.52	0.06	0.05
Heifers	1.54	1.50	0.06	0.59
Final BW, kg				
Steers	553	579	9	0.02
Heifers	575	570	9	0.66
Carcass characteristics				
Hot carcass weight, kg				
Steers	349	365	6	0.02
Heifers	362	359	6	0.66
Backfat, cm	2.46	2.40	0.14	0.76
Longissimus muscle area, cm^2^	79.6	82.4	1.2	0.03
Marbling	526	510	15	0.47
Yield grade	3.76	3.68	0.07	0.43
Carcasses grading choice or above, %	85.9	88.0	4.9	0.77

^*a*^Cows received (dry matter basis) 190 g/cow daily of Ca salts of soybean oil (CSSO; Essentiom; Church and Dwight Co., Inc., Princeton, NJ) or 170 g/cow daily of prilled saturated fat (CON; Energy Booster 100; Milk Specialties, Eden Prairie, MN). Treatments were provided from d 0 (d 195 of gestation) until calving.

Results from this experiment indicate that CSSO supplementation to late-gestating beef cows improved offspring feedlot growth, mostly in steers, and carcass LM area suggestive of programming effects on muscle development. These outcomes can also be associated with improved immunocompetence and resilience to BRD in calves from CSSO cows, given that number of BRD treatments is negatively associated with feedlot performance and carcass merit (Blakebrough-Hall et al., 2020). Accordingly, maternal nutrition impacts development of the fetal immune system and muscle tissues, including ɷ-6 FA as the CSSO source used herein (Cooke, 2019). We also expected that CSSO supplementation would improve carcass marbling, given that ɷ-6 FA stimulates adipogenesis via programming effects during gestation or early in life (Schubach et al., 2019). The benefits of CSSO supplementation during late gestation, however, were limited to muscle hypertrophy when offspring was provided a high-energy anabolic feedlot diet (Harper and Pethick, 2004), resulting in greater carcass LM area. Therefore, additional research is still warranted to understand the programming effects of ɷ-6 FA supplementation to beef cows during gestation, focusing on offspring immunocompetence, muscle and adipose tissue development, and potential impacts on hyperphagia not addressed herein (Marques et al., 2017; Cooke, 2019).

## IMPLICATIONS

Supplementing forage-fed beef cows during late gestation with CSSO did not impact cow performance during gestation, or calf growth until a 35-d preconditioning program. However, offspring from CSSO cows had improved immunocompetence in the feedlot, and expressed greater muscle development after being exposed to a high-energy feedlot diet (e.g., carcass LM area). These results are suggestive of programming effects on postnatal offspring growth and health resultant from ɷ-6 FA supplementation to late-gestating cows. Hence, supplementing CSSO to beef cows during pregnancy might be a feasible alternative to optimize offspring productivity, welfare, and carcass merit in beef production systems.



*Conflict of interest statement*. None declared.
